# Quantitative response relationships between net nitrogen transformation rates and nitrogen functional genes during artificial vegetation restoration following agricultural abandonment

**DOI:** 10.1038/s41598-017-08016-8

**Published:** 2017-08-10

**Authors:** Honglei Wang, Na Deng, Duoyang Wu, Shu Hu

**Affiliations:** State Key Laboratory of Soil Erosion and Dry land Farming on the Loess Plateau, Institute of Soil and Water Conservation, Northwest A & F University, Yangling, 712100 Shaanxi China

## Abstract

A comprehensive understanding of how microbial associated with nitrogen (N) cycling respond to artificial vegetation restoration is still lacking, particularly in arid to semi-arid degraded ecosystems. We compared soil net N mineralization rates and the abundance of bacteria, archaea, and eleven N microbial genes on the northern Loess Plateau of China during the process of artificial vegetation restoration. The quantitative relationships between net N mineralization rates and N microbial genes were determined. We observed a significant difference of net transformation rates of NH_4_^+^-N (R_a_), NO_3_^−^-N (R_d_), and total mineralization (R_m_), which rapidly decreased in 10-year soils and steadily increased in the 10–30-year soils. Different N functional microbial groups responded to artificial vegetation restoration distinctly and differentially, especially for denitrifying bacteria. Stepwise regression analysis suggested that R_a_ was collectively controlled by AOA-*amoA* and Archaea; R_d_ was jointly governed by *narG*, *napA*, *nxrA*, and bacreria; and R_m_ was jointly controlled by *napA*, *narG*, *nirK*, *nirS*, *norB*, *nosZ*, and *nxrA*.

## Introduction

Artificial vegetation restoration is an effective way to improve soil conditions and to restore degraded ecosystems, especially in the degraded ecosystems of arid to semi-arid regions^1, 2^. After water, soil N availability is the second most limiting factor for plant growth, productivity and greenhouse gas emissions in arid to semi-arid regions^2–4^. Soil N microorganisms are key drivers of ecosystem N cycling and transformation^5^. However, our understanding of N transformation and N microorganisms during the process of artificial vegetation restoration is still poor, especially in the degraded ecosystems of arid to semi-arid regions, where plant succession often exhibits a relatively rapid and predictable trajectory in terms of species diversity and composition^1, 6^.

A variety of research on vegetation restoration has emphasized on plant productivity, biomass, nutrient availability, soil structure, inter-species interaction, microbial abundance, and microbial diversity during the process of artificial vegetation restoration^7–11^. Published studies have demonstrated that the space-for-time substitution (chronosequence) is an effective way to reveal dynamic change of soil nutrient cycling and microbial communities across multiple time scales^12–14^. Nitrification, denitrification, ammonification, and N_2_ fixation are the four primary microbiological processes associated with supplying, leaching, and transforming N nutrients in soil systems^15, 16^. The three genes, ammonia-oxidizing archaea (AOA-*amoA*), ammonia-oxidizing bacteria (AOB-*amoA*), and nitrite oxidoreductase (*nxrA*), are three functional genes involved in the nitrification process (NH_4_^+^-N → NO_2_^−^-N → NO_3_^−^-N)^5^. Six other genes, periplasmic and membrane-bound nitrate reductase (*napA*/*narG*), nitrite reductase (*nirK*/*nirS*), nitric oxide reductase (*norB*), nitrous oxide reductase (*nosZ*), are six functional genes associated with denitrification (NO_3_^−^-N → NO_2_^−^-N → NO → N_2_O → N_2_)^5^. N fixation (*nifH*) is a functional gene involved in N_2_ fixation process (N_2_ → organic nitrogen)^17^. Alkaline metallopeptidases (*apr*) is a functional gene involved in ammonification (organic nitrogen → NH_4_^+^-N)^18^. Studies have focused on the general trends of microbial communities^19^, interactions among soil, plants, and microorganisms^1, 11, 20^, and potential N mineralization rates during plant restoration^2, 21^. The N cycle is a network of interlinked processes that are responsible for N fluctuations (increases and losses) by increasing NH_4_^+^-N (N_2_ → NH_4_^−^-N), the leaching of NO_3_^−^-N and NO, and N_2_O or N_2_ emissions caused by ammonification, N_2_-fixation, nitrification, and denitrification^5, 16, 22^. However, relatively few studies have focused on soil microbial properties and N functional microbes during the process of artificial vegetation restoration, and very little is known in regard to the fate of N processing and the underlying mechanism that governs the N transformation.

The northern part of the Chinese Loess Plateau is a region of traditional agriculture and pastoral land use that suffers from extensive and severe water- and wind-driven soil erosion^2^. For the past 40 years, abandoning sloped farmland and artificial afforestation are the most frequently used practices of preventing soil erosion and rehabilitating ecological environments on the plateau^23, 24^. The effects of artificial afforestation on soil nutrient properties, bacterial, and fungal dynamics have been reported^11, 25, 26^, but very little is known regarding the shift of soil net N transformation rates, N functional microbes, and underlying N transformation mechanisms. This information can help achieve in-depth understanding N cycling process, reactive N availability and N_2_O emissions potential during ecological restoration, providing predictions and mitigation strategies for N_2_O emissions.

Therefore, we investigated the dynamics of soil N transformation rates and N functional microbes at sites representing 40 years of artificial afforestation in abandoned farmland on the Loess Plateau. The objective of this study was to: (1) evaluate net N transformation rates during the 40 years of forest ecosystem restoration after agricultural abandonment; (2) quantify the dynamic evolution of N microbial genes in the process of vegetation restoration; (3) determine quantitative relationships between net N transformation rates and N functional genes; and (4) discern key functional genes that govern net N transformation.

## Results

### Vegetation and soil characteristics

This study was conducted with plant and soil samples during artificial vegetation restoration following agricultural abandonment, and the sites included cultivated soils (0-y) and uncultivated soils (10-y, 20-y, 30-y, and 40-y, respectively). The crops at the 0-y sites were harvested, and the plant cover were not quantified. *Robinia pseudoacacia* cover ranged from 42.5% at 10-y sites to 67.5% at 30-y sites (Table S1), and the undergrowth herbaceous vegetation significantly ranged from 31.4% at 10-y sites to 55.60% at 30-y sites (Table S2).

Significant differences in soil characteristics were found between sites as vegetation restoration progressed (Table 1). The contents of soil organic C and total N showed a similar trend, and significantly increased at 0–40-year sites (ANOVA with Tukey’s HSD test, *P* < 0.05, *n* = 15). The contents of NO_3_^−^-N at the 10-y sites decreased steadily compared to the 0-y sites and then increased significantly with increasing site age (ANOVA with Tukey’s HSD test, *P* < 0.05, *n* = 15). The contents of NO_3_^−^-N at the 10-y sites decreased steadily compared to the 0-y sites and then increased significantly at 20–40-year sites (ANOVA with Tukey’s HSD test, *P* < 0.05, *n* = 15). Bulk densities significantly decreased at the 0–40-year sites (ANOVA with Tukey’s HSD test, *P* < 0.05, *n* = 15). Soil pH ranged from 8.47 to 8.73 between the 0–40-year sites.

**Table 1:** Soil physicochemical properties during the process of artificial vegetation restoration.

Plots	0-y	AVR-10-year	AVR-20-year	AVR-30-year	AVR-40-year
Organic C (g kg^−1^)	3.27 ± 0.12e	3.69 ± 0.42d	4.09 ± 0.18c	5.03 ± 0.36b	8.52 ± 1.27a
Total N (g kg^−1^)	0.52 ± 0.06d	0.54 ± 0.02c	0.54 ± 0.06bc	0.57 ± 0.03b	0.88 ± 0.11a
NO_3_^–^N (mg kg^−1^)	4.23 ± 0.09d	4.18 ± 0.16bc	4.19 ± 0.20c	4.46 ± 0.10b	5.21 ± 0.13a
NH_4_^+^-N (mg kg^−1^)	9.93 ± 0.72b	9.73 ± 0.63c	13.26 ± 0.25a	8.29 ± 0.18d	7.97 ± 0.07e
pH	8.47 ± 0.02c	8.70 ± 0.02a	8.73 ± 0.03a	8.72 ± 0.02a	8.55 ± 0.15b
Bulk density (g cm^−1^)	1.30 ± 0.01a	1.23 ± 0.01b	1.12 ± 0.00c	1.04 ± 0.04d	0.94 ± 0.02e
Water content (%)	18.25 ± 0.04a	16.86 ± 0.11b	12.26 ± 0.04e	12.41 ± 0.03d	14.67 ± 0.05c

Values are means ± standard error (n = 3). AVR: artificial vegetation restoration. Capital letters denote significant differences between sites (*P* < 0.05, ANOVA with Tukey’s HSD) for each variable.

### Soil net N transformation rates

**Figure 1:**
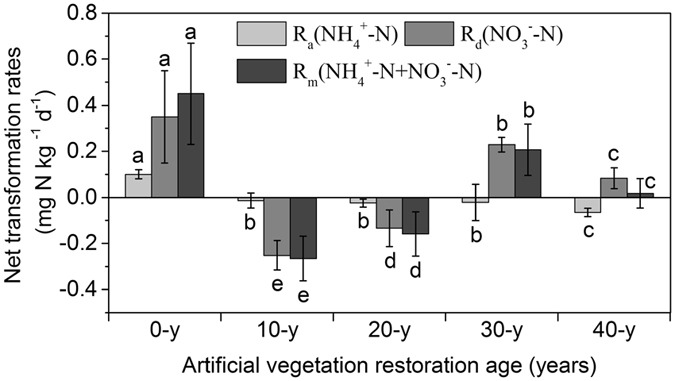
Net transformation rates of R_a_ (NH_4_^+^-N), R_d_ (NO_3_^−^-N), and total mineralization (R_m_) during artificial vegetation restoration. Values are means ± standard error (n = 3). Different letters indicate significant differences (*P* < 0.05) among soils for the individual variables based on a one-way ANOVA followed by an LSD test.

R_a_ (NH_4_^+^-N), R_d_ (NO_3_^−^-N), and R_m_ (NH_4_^+^-N + NO_3_^−^-N) were different from each other during the process of artificial vegetation restoration (ANOVA with Tukey’s HSD test, *P* < 0.05, *n* = 15) (Fig. 1). R_a_ steadily decreased during the 40 years of soil recovery compared with that at the 0-y sites, ranging from −0.013 at 10-y sites to −0.065 at 40-y sites mg N kg^−1^d^−1^. R_d_ and R_m_ first decreased markedly at the 0-y sites but then steadily increased during the 30 years of soil recovery compared with that at the 0-y sites, showing the highest values of 0.228 and 0.206 mg N kg^−1^ d^−1^ at 30-y sites, respectively.

### Abundance of genes

**Figure 2:**
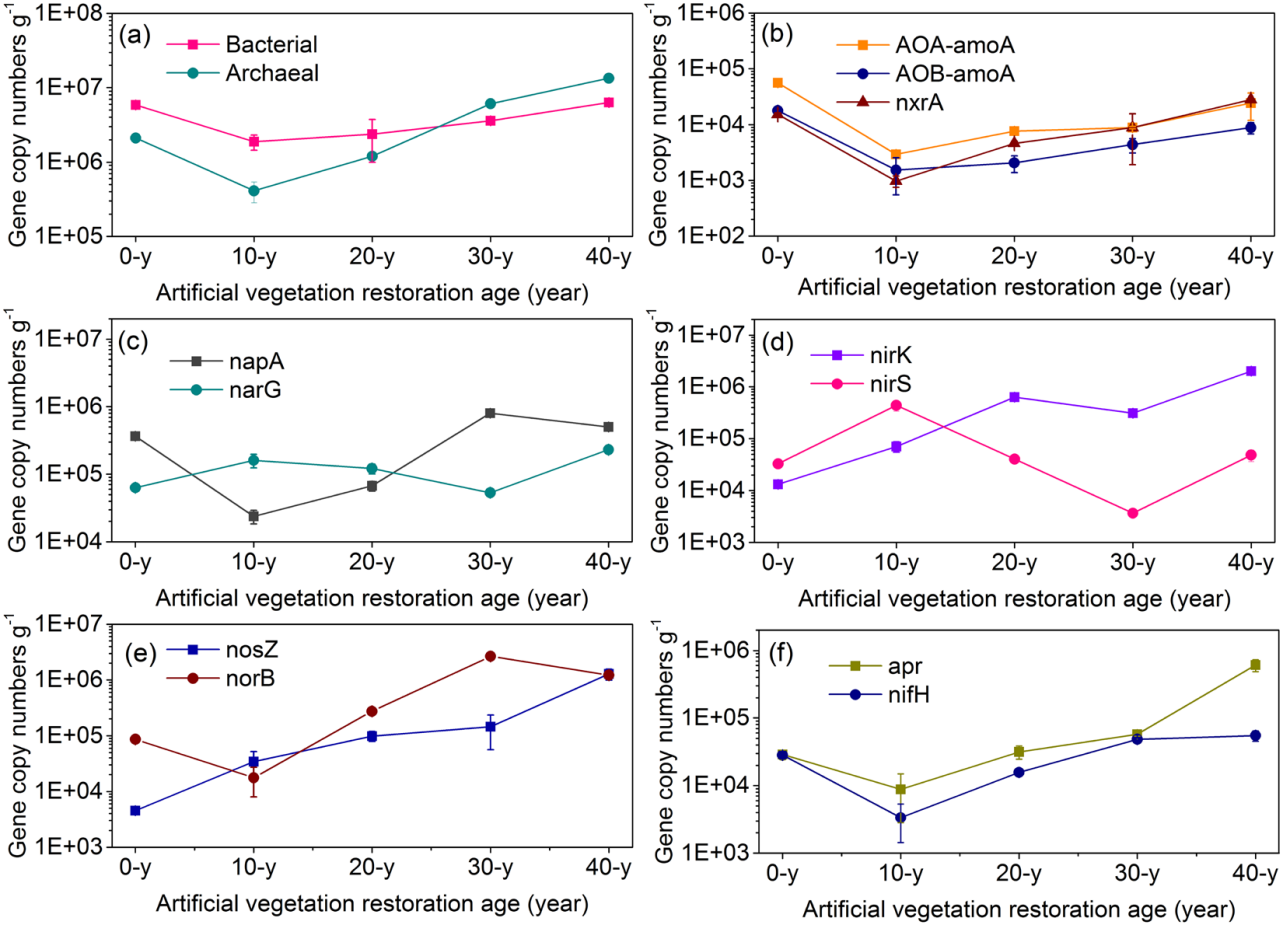
The absolute abundances of bacteria, archaea, and nitrogen functional genes during artificial vegetation restoration. (**a**) Bacterial and archaeal 16S rRNA; (**b**) AOA-*amoA*, AOB-*amoA*, and *nxrA*; (**c**) *narG* and *napA*; (**d**) *nirK* and *nirS*; (**e**) *nosZ* and *norB*; and (**f**) *apr* and *nifH*. The absolute abundances (copies g^−1^) are shown on log10 scale (Y-axis). Standard deviations of three replicates are indicated by error bars. Invisible error bars indicate that the standard deviations are smaller than the marker size.

The absolute abundance of bacteria, archaea, and N functional genes varied along with ages in the process of vegetation restoration (Fig. 2). Across all sites, bacteria, archaea, AOA-*amoA* and AOB-*amoA*, *nxrA*, *apr*, and *nifH* genes showed a similar evolutionary tendency, with abundance initially decreasing compared with that at the 0-y sites, followed by a similar increased pattern at the 10–40-year sites. The AOA-*amoA* gene was (1.8–3.7 times) higher than AOB-*amoA* gene. Across all sites, *napA*, *narG*, *nirK*, and *nirS* genes exhibited an adverse fluctuation tendency. The *napA* gene ranged from 2.38 × 10^4^ at 10-y sites from 8.05 × 10^5^ copy numbers g^−1^ at 30-y sites. The *narG* gene ranged from 5.29 × 10^4^ at 30-y sites from 2.32 × 10^5^ copy numbers g^−1^ at 40-y sites. The *nirK* gene ranged from 1.32 × 10^4^ at 0-y sites from 2.03 × 10^6^ copy numbers g^−1^ at 20-y sites. The *nirS* ranged from 3.67 × 10^3^ at 0-y sites from 4.40 × 10^5^ copy numbers g^−1^ at 10-y sites. The *nosB* slightly decreased from 8.59 × 10^4^ at 0-y sites to 1.76 × 10^4^ copy numbers g^−1^ at 10-y sites but then reached a final high value of 1.44 × 10^5^ copy numbers g^−1^ at 30-y sites. The *nosZ* steadily increased from 4.53 × 10^3^ at 0-y sites to 1.26 × 10^6^ copy numbers g^−1^ at 40-y sites.

### Relative abundance and richness

**Figure 3:**
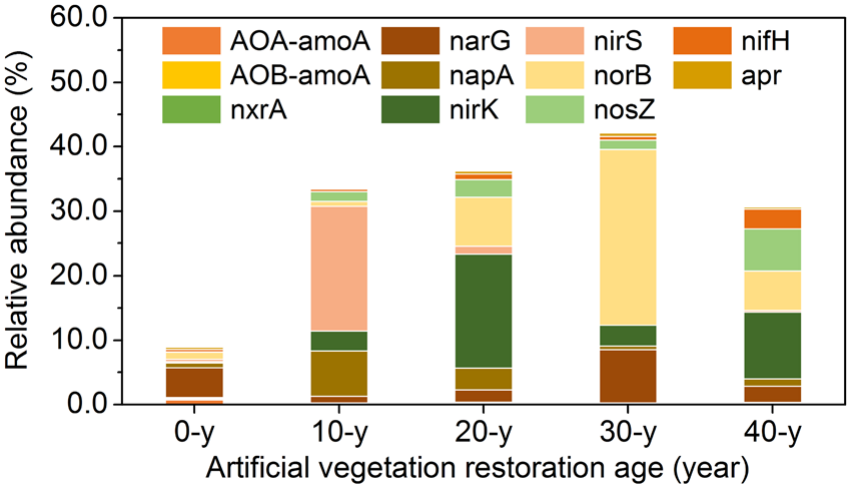
The relative abundance of nitrogen functional genes during artificial vegetation restoration. (The relative abundance was defined as the percentage of absolute abundance of a nitrogen functional gene divided by the absolute abundance of bacteria and archaea).

**Figure 4:**
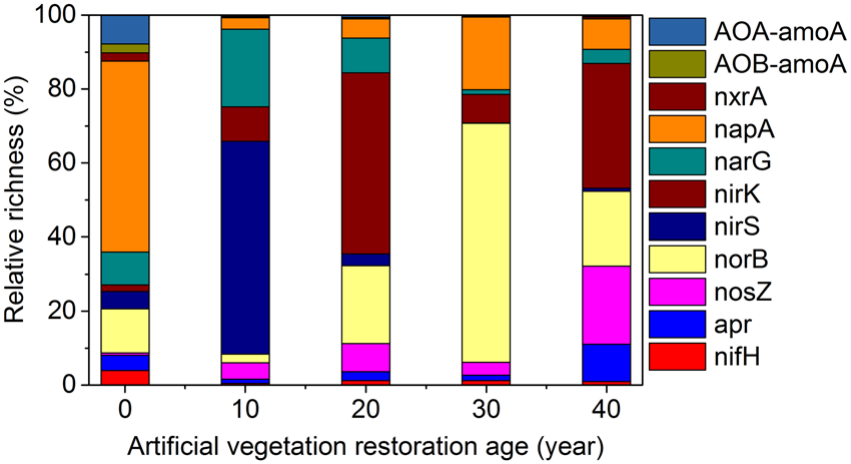
The relative richness of nitrogen functional genes during artificial vegetation restoration. (The relative richness was defined as the percentage of absolute abundance of a nitrogen functional gene divided by the absolute abundance of all nitrogen functional genes).

The relative abundance of N functional genes (relative to bacteria and archaea) steadily increased from 8.92% (0-y sites) to 42.11% (10-y sites), but then decreased to 30.59% (40-y sites) (Fig. 3). The dominant N functional genes varied along the restoration chronosequence (Fig. 4). The relative richness of *napA* (51.59%), *norB* (12.02%),*narG* (8.87%),and *AOA*-*amoA* (7.81%) was significantly higher than that at the 0-y sites, which suggested that *napA*, *norB*, *narG*, and *AOA*-*amoA* were the dominant N functional genes at the 0-y sites. *nirS* (57.48%), *narG* (21.02%), *nirK* (9.30%), and (4.49%) were the dominant N functional genes at the 10-y sites. *nirK* (48.93%), *norB* (21.07%), *narG* (9.36%), and *nosZ* (7.59%) were the dominant N functional genes at the 20-y sites. *norB* (64.60%), *napA* (19.69%), and *nirK* (7.66%) were the main N functional genes at the 30-y sites. *nirK* (33.75%), *nosZ* (21.14%), *norB* (20.19%), and *apr* (10.08%) were the key N functional genes at the 40-y sites.

### Correlations between functional genes and soil properties

**Figure 5:**
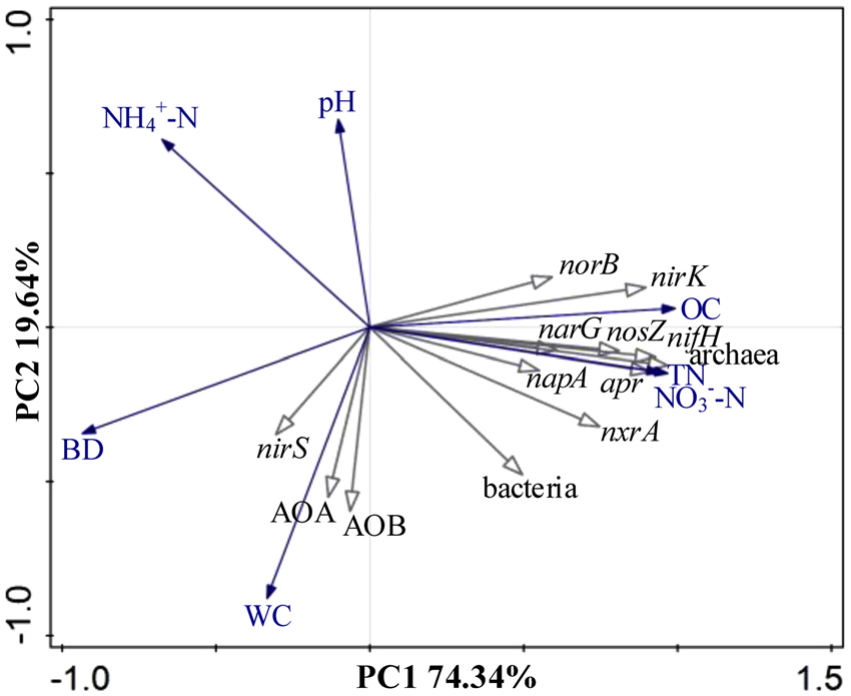
Principal component analysis of soil properties (Bulk density (BD), pH, total nitrogen (TN), organic carbon (OC), NH_4_^+^-N, NO_3_^−^-N, water content (WC), bacterial 16S rRNA (bacteria), archaeal 16S rRNA (archaea), and eleven N functional genes (i.e., AOA-*amoA*, AOB-*amoA*, *nxrA*, *narG*, *napA*, *nirK*, *nirS*, *norB*, *nosZ*, *apr*, and *nifH*), along the artificial vegetation restoration. The first two PCA axes explain 74.34% of total variance.

Ordination of samples by PCA based on soil properties, bacteria, archaea, and eleven N functional genes (i.e., AOA-*amoA*, AOB-*amoA*, *nxrA*, *narG*, *napA*, *nirK*, *nirS*, *norB*, *nosZ*, *apr*, and *nifH*) showed a clear separation of vegetation restoration stages along the first axis (Fig. 5), with the first two axes explaining 74.34% of total variance. We found positive correlations among organic carbon, total nitrogen, NO_3_^−^-N, bacteria, archaea, *nxrA*, *narG*, *napA*, *nirK*, *norB*, *nosZ*, *apr*, and *nifH*. pH and NH_4_^+^-N were negatively correlated with AOA-*amoA*, AOB-*amoA*, and *nirS*.

### Quantitative relationships

Eleven functional genes, (i.e. AOA-*amoA*, AOB-*amoA*, *nxrA*, *narG*, *napA*, *nirK*, *nirS*, *norB*, *nosZ*, *apr*, and *nifH* absolute abundance) were employed as candidate variables in stepwise regression analysis to associate with R_a_, R_d_, and R_m_. Results showed R_a_ equation was successfully established (R_a_ = 6.398 × 10^−6^ AOA-*amoA* – 0.011, *R* ^2^ = 0.823, *P* = 0.021). For example, the R_a_ was estimated 6.4-fold greater when AOA-*amoA* gene abundance increased from 1.0 × 10^−6^ to 1.0 × 10^−7^ copy numbers g^−1^. However, low *R* ^2^ values (*R* ^2^ = 0.823) and absence of comprehensive interpretations for the equation spurred us to carefully re-examine the stepwise regression analysis.

By introducing a series of reasonable variables in stepwise regression analysis, all three R_a_, R_d_, and R_m_ equations were successfully established with higher *R* ^2^ values ranging from 0.928 to 0.995 (Table 2). In the improved stepwise regression models, R_a_ was determined from AOA-*amoA*/Archaea. R_d_ was jointly determined from *narG*/bacreria and *nxrA*/*napA*. R_m_ was jointly determined from *nxrA*/(*nirK* + *nirS*) and (*napA* + *narG*)/(*napA* + *narG* + *nirK* + *nirS* + *norB* + *nosZ*).

**Table 2:** Quantitative response relationships between net N transformation rates (mg N kg^−1^ d^−1^) and functional gene abundance (copies g^−1^) during the process of artificial vegetation restoration.

Stepwise regression models (equations)	*F*	*R* ^2^	*P value*
${{\rm{R}}}_{{\rm{a}}}=5.825\frac{AOA-amoA}{Archaea}-0.055$	38.83	0.928	0.008
${{\rm{R}}}_{{\rm{d}}}=7.950\frac{narG}{bacreria}-5.746\frac{nxrA}{napA}+0.590$	202.17	0.995	0.005
${{\rm{R}}}_{{\rm{m}}}=3.717\times {10}^{-7}\frac{(napA+narG)}{(napA+narG+nirK+nirS+norB+nosZ)}-2.096\frac{nxrA}{(nirK+nirS)}-0.253$	73.078	0.987	0.013

## Discussion

### Nitrogen transformation mechanisms

As artificial vegetation restoration proceeded, the abundance of bacteria and archaea first decreased compared with that at 0-y sites but then steadily increased in the 10-, 20-, 30- and 40-y soils (Fig. 2a). The bacteria and archaea exhibited a similar temporal variation trend with soil nutrient accumulation (Table 1). This finding is supported by previous studies showing that the increasing in vegetation cover and soil nutrients along a chronosequence have a positive on-going impact on the enhancement of the bacterial and archaeal community^11, 26, 27^.

The AOA-*amoA*, AOB-*amoA*, and *nxrA* are the three functional genes involved in NH_4_^+^-N transformation (NH_4_^+^-N → NO_2_^−^-N → NO_3_^−^-N). All three genes exhibited decreases in 10-y sites compared with that at the 0-y sites but then steadily increased in the 10–40-year soils (Fig. 2b). The AOA-*amoA*, AOB-*amoA*, and *nxrA* exhibited increases in the 10–40-year soils, leading to enhanced nitrifying activity responsible for eliminating NH_4_^+^-N and increasing NO_3_^−^-N. The abundance of AOA-*amoA* at all sites was 1.8–3.7 times greater than that of AOB-*amoA*, and most studies of ammonia oxidizers in terrestrial ecosystems have found that AOA is more abundant than AOB and AOA play a major role in determining soil NH_4_^+^-N transformation (NH_4_^+^-N → NO_2_^−^-N)^28, 29^. Studies have suggested that niche partitioning occurs between AOA and AOB, with ammonia concentrations and soil pH representing the main environmental factors shaping the ecological niches of ammonia oxidizers^30, 31^. In the present study (arid and semiarid ecosystems), all investigated sites showed a similar spatial distribution of AOA and AOB, which suggests coexistence of the two groups of ammonia oxidizers. The five sites were characterized by low ammonia concentrations (7.97–13.26 mg N kg^−1^) with small variations in pH (8.47–8.73) (Table 1). Because the soil properties (i.e., NH_4_^+^-N, pH, and organic C) in 0–40-year soils did not separate the niches of AOA and AOB (Fig. 5), we suggest that factors that are otherwise masked by gradients in NH_4_^+^-N or pH are the primary reason accounted for the coexistence of AOA and AOB. The AOA-*amoA* and AOB-*amoA* gene showed a similar temporal variation trend to the *nxrA* gene. This associated pattern of fluctuation was due to similar environmental adaptations and ecological interactions between AOA, AOB and nitrite-oxidizing bacteria (NOB)^29^. The *nifH* and *apr* genes are two functional genes involved in NH_4_^+^-N production (N_2_ → NH_4_^+^-N). The two functional genes exhibited increases in the 10–40-year soils (Fig. 2f), leading to enhanced N_2_ fixing and ammonifying activity responsible for increasing NH_4_^+^-N. Soil organic carbon is a key factor that affect the abundance of *nifH* and *apr* genes in soils^15, 32^. This finding is supported by previous studies showing that the increasing in soil organic carbon have a positive on-going impact on the enhancement of the *nifH* and *apr* ^33^. Furthermore, results of the integrated analysis show that the ratio (*nifH* + *apr*)/(AOA-*amoA* + AOB-*amoA*), indicating NH_4_^+^-N accumulation, steadily increased in the 10–40-year soils (Fig. S1a). This pattern might explain the corresponding R_a_ (NH_4_^+^-N) accumulation in the 10–40-year soils (Fig. 1).

*napA*, *narG*, *nirK*, *nirS*, *norB*, and *nosZ* are the six functional genes involved in denitrification processes. The abundance of *napA* and *narG* exhibited different fluctuating trends along with ages in the process of vegetation restoration (Fig. 2). These results agree with previous research showing that the *napA* and *narG* genes display mutual inhibition and that *narG* is easily promoted by increases in soil nutrients^22, 34^. Furthermore, results of the integrated analysis show that the ratio *nxrA*/(*napA* + *narG*), indicating NO_3_^−^-N accumulation, steadily increased in the 10–40-year soils (Fig. S1b). This pattern might explain the corresponding R_a_ (NO_3_^−^-N) fluctuating trends in the 10–40-year soils (Fig. 1). The abundance of *nirK* and *nirS* varied greatly along the 40-year vegetation restoration, which suggested that *nirK*- and *nirS*-type bacteria choose different habitats after substantial variations in soil physicochemical properties. This notion is supported by previous studies showing that the niches of these two types of *nir*-harboring bacteria are responsible for their different behaviors^22, 35^. A significant increase in *norB* gene abundance (absolute abundance, relative abundance and richness) was observed in 10–30-year soils (Figs 2e and 3, and Fig. S1c), leading to continuous increase of NO emission (not measured in this study). A steady increase in *nosZ* gene abundance was observed as artificial vegetation restoration progressed. This increase in *nosZ* during long-term vegetation restoration enhanced the last step in the denitrification pathway, leading to potential increase of R_a_ and R_m_.

### Quantitative Response Relationships

Across the soils from the five different plant communities used in this study, we found the AOA-*amoA* gene was the rate-limiting genes that solely determined the R_a_, consistent with the results by Caffrey, *et al*.^36^, suggesting that the first and rate-limiting step in nitrification is catalyzed by the enzyme ammonia monooxygenase. Although the single nitrogen transformation process and the underlying functional genes that drive the cycling are well understood, our knowledge of the roles of these functional genes in nitrogen transformation is still descriptive. Therefore, quantitative response relationships were developed to link macro-scale nitrogen processes and microscale functional genes and to advance our quantitative understanding of the key genes that govern the nitrogen transformation processes.

In improved stepwise regression models, R_a_ (NH_4_^+^-N) was jointly determined by AOA-*amoA* and Archaea. The variable AOA-*amoA*/Archaea, indicating NH_4_^+^-N oxidation, showed a positive relationship with net NH_4_^+^-N transformation rates, because both AOA -*amoA* gene and Archaea were primarily associated with NH_4_^+^-N conversion^5, 31^. Thus, the increasing AOA-*amoA* and Archaea genes with were the key factors responsible for losing NH_4_^+^-N as artificial vegetation restoration progressed.

R_d_ (NO_3_^−^-N) was jointly determined by *narG*, *napA*, and *nxrA* (Table 2). The first variable *narG*/bacreria in the equation denotes the transformation levels of NO_3_^−^-N, and this variable’s positive correlation with the R_d_ is in agreement with previous studies that reported the high ratio of this variable represents the extent of NO_3_^−^-N reduction^4^. The second variable *nxrA*/*napA*, indicating NO_3_^−^-N accumulation, showed a negative relationship with R_d_ (Table 2). The *nxrA* gene was involved in NO_3_^−^-N production^5^, while *napA* gene was involved in NO_3_^−^-N consumption^5^, therefore the production and consumption ratio symbolized the extent or level of NO_3_^−^-N accumulation (increased), which is the opposite of NO_3_^−^-N transformation (decreased) in terms of reaction direction. This result suggests that the nitrifying gene *nxrA* may play an underlying, but previously unrecognized, role in the denitrification process and nitrogen reduction. This functional interaction between the nitrifying and denitrifying communities may alter our traditional perspective that the nitrification and denitrification process, which requires different conditions, are functionally independent and separate^37^.

R_m_ was jointly determined by *narG*, *napA*, *nirK*, *nirS*, *norB*, *nosZ*, and *nxrA*. The variable (*napA* + *narG*)/(*napA* + *narG* + *nirK* + *nirS* + *norB* + *nosZ*) was identified as the relative share of NO_3_^−^-N reduction in denitrification process (Table 2). The high ratio of this variable represents the extent of NO_3_^−^-N reduction, supporting our above analyses. The variable *nxrA*/(*nirS* + *nirK*), indicating NO_2_^−^-N transformation, showed a negative relationship with R_m_ transformation. The *nxrA* gene is involved in NO_2_^−^-N consumption (NO_2_^−^-N → NO_3_^−^-N), and *nirK* and *nirS* are involved in NO_2_^−^-N consumption^5^. Therefore, the consumption ratio represents the extent of NO_3_^−^-N production, and the more the NO_2_^−^-N accumulation, the greater the NO_3_^−^-N production.

Our results exhibited the ratio of N functional genes and relative abundance rather than the absolute abundances of N functional genes were the primary reason accounted for the quantitative relationship with the net NH_4_^+^-N and NO_3_^−^-N transformation, consistent with the findings by previous studies^38, 39^, suggesting that the abundances of N functional genes (groups) were the variables that best explained the variation in net N transformation. Absolute abundance data are of primary importance in microbial studies and are routinely employed to determine genes of interest and quantify the exact copy number in the environment^40^. Relative abundance might be more available for quantifying the dynamics of the ecological processes being carried out, which are affected by a number of microbial groups^38, 41^.

## Materials and Methods

### Description of sites and sample collection

The experimental sites (109°15′E, 36°44′N) were established in the Zhifanggou Ecological Restoration Watershed on the Loess Plateau (109°15′E, 36°44′N). The region has semi-arid continental climate with a mean annual temperature of 8.8 °C and a mean minimum temperature in January of 6.2 °C and a mean maximum temperature in August of 37.2 °C. The mean annual precipitation is approximately 510 mm with approximately 73.6% of the annual precipitation distributed during the growing season (July to September). The soil is classified as a Huangmian soil (a Calcaric Cambisol in the FAO Soil Classification System). The dominant planted tree species in the study area is *Robinia pseudoacacia*.

We used the chronosequence method to evaluate the response of the soil bacteria, Archaea, and N functional communities to the artificial vegetation restoration of abandoned farmland. Five stages (0, 10, 20, 30, and 40 years) of vegetation restoration can be observed in the watershed subjected to ecological restoration. A total of 12 abandoned farmland areas at the 10-, 20-, 30-, and 40-y stages of restoration were selected as the experimental sites. Three active sloped farmland areas growing corn were used as a baseline or control (0 year).

### Soil chemical parameters

The undisturbed buried core method was used to measure the *in situ* net N mineralization^42, 43^. In 20 July 2016, we randomly selected three sharpened PVC cores (5 cm diameter × 22 cm long) in each site, and were driven 20 cm into the ground and covered with permeable plastic film to prevent water penetration and allow gas exchange during core incubation^2^. Soil samples were collected from 0 to 20 cm to determine the initial conditions. After the cores were incubated in the sites for 26–30 days each, the cores were transported to the laboratory and stored at 4 °C prior to processing.

Soil moisture was determined gravimetrically in fresh soils at 105 °C overnight, and the water content was expressed as a percentage of the dry weight. Soil bulk density, organic carbon, total phosphorus, available phosphorus, nitrate (NO_3_^−^-N), and ammonium (NH_4_^+^-N) were measured using methods described previously^44^. The soil pH was determined using a glass electrode meter in 1:2.5 (soil: water) suspensions. Because the concentration of nitrite (NO_2_^−^-N) in the soils was negligible, only NO_3_^−^-N and NH_4_^+^-N were determined.

### Quantitative polymerase chain reaction (qPCR)

Total genomic DNA was extracted by D5625–01 soil DNA kit (Omega, USA). Quantitative analysis was conducted for fragments of the bacterial 16S rRNA, archaeal 16S rRNA, and eleven N functional genes (i.e., AOA-*amoA*, AOB-*amoA*, *nxrA*, *narG*, *napA*, *nirK*, *nirS*, *norB*, *nosZ*, *apr*, and *nifH*). qPCR was performed in a CFX Real-Time PCR Detection System (Bio-Rad, USA) via a three-step thermal cycling procedure, with a 20 µL reaction mixture consisting of 10 µL SYBR Green I PCR master mix (Applied Biosystems, USA), 1 µL template DNA (sample DNA or plasmid DNA for standard curves), 1 µL forward and reverse primers, and 7 µL sterile water (Millipore, USA). The protocol and parameters for each target gene are presented in Table S3. The *R* ^2^ value for each standard curve exceeded 0.99, which indicated linear relationships across the concentration ranges used in this study.

### Statistical analysis

The net transformation rates of NH_4_^+^-N (R_a_), NO_3_^−^-N (R_d_), and total mineralization (R_m_) during the incubation period were calculated from the difference between the initial and final concentrations of NH_4_^+^-N, NO_3_^−^-N, and total mineral N (NH_4_^+^-N, NO_3_^−^-N). The standard deviations (S.Ds.) of the gene abundance data were calculated using three replicates measured via qPCR and plotted as error bars for the assessment of variations in the data and measurement errors. A principal component analysis was applied to investigate the response of net N transformation rates to soil properties using CANOCO software 4.5. Stepwise regression analysis were built to determine the multiple linear regression equation between net N transformation rates and N functional genes (63 functional gene groups associated with nitrogen transformation, see Table S4) using SPSS Statistics 20 (IBM, USA).

## Electronic supplementary material

Supplementary information
