# Supplementary material for: Quantitative response relationships between net nitrogen transformation rates and nitrogen functional genes during artificial vegetation restoration following agricultural abandonment
Source: Sci Rep. 2017 Aug 10;7:7752. doi: 10.1038/s41598-017-08016-8 (PMC5552692; doi:10.1038/s41598-017-08016-8)
Supplement: Supplementary file 1 — Supplementary information [file 41598_2017_8016_MOESM1_ESM.pdf]

Quantitative response relationships between net nitrogen transformation rates and nitrogen functional genes during artificial vegetation restoration following agricultural abandonment

Honglei Wang*, Na Deng, Duoyang Wu, Shu Hu

State Key Laboratory of Soil Erosion and Dry land Farming on the Loess Plateau, Institute of Soil and Water Conservation, Northwest A & F University, Yangling 712100, Shaanxi, China

*Corresponding author. E-mail addresses: wanghonglei@nwsuaf.edu.cn (HL. W). Tel.: +86 02987012411; fax: +86 02987012210

Table S1 Characteristics of the sampling sites.

Site name	Longitude	Latitude	Slope aspect	Slope (°)	Altitude (m)	RP cover (%)
0-y [a]	109.27° E	36.73° N	E25°S	18-23	1260	-
	109.27° E	36.73° N	E25°S	10-18	1259	-
	109.27° E	36.73° N	E25°S	10-20	1260	-
AVR-10-year	109.25° E	36.72° N	S30°N	26-33	1312	50-55
	109.25° E	36.72° N	E38°S	31-42	1320	30-40
	109.25° E	36.73° N	E30°W	22-37	1302	30-50
AVR-20- year	109.24° E	36.72° N	E31°S	28-32	1281	30-40
	109.26° E	36.74° N	W21°S	18-26	1289	45-60
	109.26° E	36.74° N	W25°S	20-30	1278	45-50
AVR-30- year	109.25° E	36.75° N	S33°N	30-38	1197	50-60
	109.25° E	36.75° N	S26°N	24-30	1205	70-80
	109.25° E	36.75° N	S31°N	24-38	1216	70-75
AVR-40-year	109.25° E	36.75° N	W30°S	27-34	1126	35-40
	109.25° E	36.75° N	W34°S	26-34	1140	45-50
	109.25° E	36.76° N	W26°S	24-29	1151	40-50

[a] Coverage was not taken in to account because the farmland was harvested; AVR: artificial vegetation restoration. RP: *Robinia pseudoacacia.*

Table S2 Vegetation coverage during the artificial vegetation restoration of abandoned farmland.

Coverage (%)	0-y *	AVR-10-y	AVR-20-y	AVR-30-y	AVR-40-y
Cleistogenes squarrosa	-	1.4 ± 0.04	-	-	-
Bidens parviflora	-	27.2 ± 0.91	-	-	-
Sonchus oleraceus	-	0.2 ± 0.00	-	-	-
Erodium stephanianum	-	0.1 ± 0.00	-	-	-
Salsola collina	-	0.1 ± 0.00	1.8 ± 0.04	-	-
Heteropappus altaicus	-	0.6 ± 0.02	-	-	0.2 ± 0.00
Stipa bungeana	-	0.6 ± 0.02	1.2 ± 0.03	2.2 ± 0.05	2.2 ± 0.23
Melica scabrosa	-	1.2 ± 0.04	1.6 ± 0.04	1.2 ± 0.02	0.2 ± 0.01
Artemisia argyi	-	-	0.2 ± 0.00	-	-
Leymus secalinus	-	-	0.2 ± 0.00	-	-
Artemisia mongolica	-	-	1.2 ± 0.01	-	-
Rubia cordifolia	-	-	0.2 ± 0.00	-	-
Leonurus artemisia	-	-	0.4 ± 0.01	-	-
Dracocephalum moldavica	-	-	0.4 ± 0.00	-	-
Clematis fruticosa	-	-	1.0 ± 0.03	-	1.0 ± 0.06
Artemisia sacrorum	-	-	36.4 ± 2.08	33.1 ± 2.52	24.0 ± 1.84
Cleistogenes chinensis	-	-	1.0 ± 0.01	0.2 ± 0.03	0.2 ± 0.02
Periploca sepium	-	-	-	0.3 ± 0.01	-
Ziziphus jujuba	-	-	-	0.5 ± 0.03	-
Solanum septemlobum	-	-	-	4.7 ± 0.49	-
Lespedeza daurica	-	-	-	0.2 ± 0.00	0.6 ± 0.03
Rubus parvifolius	-	-	-	13.3 ± 1.06	6.6 ± 0.58
Syringa oblata	-	-	-	-	0.2 ± 0.01
Glycyrrhiza uralensis	-	-	-	-	0.2 ± 0.02
Artemisia leucophylla	-	-	-	-	0.8 ± 0.08
Potentilla tanacetifolia	-	-	-	-	0.4 ± 0.02
Serratula centauroides	-	-	-	-	0.4 ± 0.03
Total	-	31.4 ± 1.65 d	45.6 ± 4.02 b	55.6 ± 3.86 a	37.0 ± 5.13 c

* The crops on the farmland were harvested, and the plant characteristics were not measured in this study. Data are mean ± standard error (n =3). Different letters indicate significant differences ($P < 0.05$) among soils for the individual variables based on a one-way ANOVA followed by an LSD test.

Table S3 Primers and thermal profiles used for real-time PCR quantification of the different phylogenetic and functional genes.

Target gene	Primers	Primer sequence (5'-3')	*bp*	Thermal profile	References
Bacteria 16S rRNA	Eub338	ACT CCT ACG GGA GGC AGC AG	365	95°C 5 min; 95°C 60 s, 53°C 30	1
	Eub518	ATT ACC GCG GCT GCT GG		s, 72°C 60 s; 35 cycles	
Archaeal 16S rRNA	Ar109f	ACKGCTCAGTAACACGT	235	95°C 5 min; 95°C 45 s, 54°C 40	2
	Ar344r	TCGCGCCTGCTGCTCCCCGT		s, 72°C 45s; 35 cycles	
AOA-*amoA*	CamoA-19F	ATGGTCTGGYTWAGACG	629	95°C 5 min; 95°C 30 s, 50°C 60	3
	CamoA-616R	GCCATCCABCKRTANGTCCA		s, 72°C 60 s; 35 cycles	
AOB–*amoA*	amoA-1F	GGGGTTTCTACTGGTGGT	491	94°C 5 min; 94°C 30 s, 55°C 45	4
	amoA-2R	CCCCTCKGSAAAGCCTTCTTC		s, 72°C 60 s; 40 cycles	
nxrA	nxrA-F1norA	CAGACCGACGTGTGCGAAAG	322	95°C 10 min, 95°C 15 s, 56.5°C	5
	nxrA-R1norA	TCYACAAGGAACGGAAGGTC		30 s, 72°C 30 s; 40 cycles	
napA	napA V17F	TGGACVATGGGYTTYAAYC	152	95°C 10 min, 95°C 15 s, 56.5°C	6
	napA 4R	ACYTCRCGHGCVGTRCCRCA		45 s, 72°C 30 s; 40cycles	
narG	narG-1960m2f	TA(CT)GT(GC)GGGCAGGA(AG)AAACTG	100	95°C 10 min; 95°C 15 s, 58°C	7
	narG-2050m2r	CGTAGAAGAAGCTGGTGCTGTT		45 s, 72°C 30 s; 40cycles	
nirK	nirK583F	TCA TGGTGCTGCCGCGKGACGG	326	95°C 10 min; 95°C 15 s, 64°C	8
	nirK909R	GAA CTTGCCGGTKGCCCAGAC		45 s, 72°C 30 s; 40cycles	
nirS	*nirS*cd3aF	GT(C, G)AACGT(C, G)AAGGA(A, G)AC(C, G)GG	425	95°C 10 min; 95°C 15 s, 64°C	9
	*nirS*R3cd	GA(C, G)TTC GG(A, G) TG(C, G)GTCTTG A		45 s, 72°C 30 s; 40cycles	
norB	*norB*2F	GGNCAYCARGGNTAYGA	262	95°C 10 min; 95°C 15 s, 57.5°C	10
	*norB*5R	ACCCANAGRTGNACNACCCACCA		50 s, 72°C 30 s; 40cycles	
nosZ	*nosZ* 1527F	CGCTGTTCHTCGACAGYCA	250	95°C 10 min; 95°C 15 s, 58°C	11
	nosZ 1773R	ATRTCGATCARCTGBTCGTT		50 s, 72°C 30 s;40cycles	
nifH	nifH F	AAAGGYGGWATCGGYAARTCCACCACTT	460	95°C 10 min; 95°C 15 s, 54°C	12
	nifH R	GTTSGCSGC R TACATSGCCATCAT		50 s, 72°C 30 s; 40cycles	
apr	*apr F*	*TAYGGBTTCAAYTCCAAYAC*	233	95°C 5 min; 94°C 30 s, 53°C 30	13
	apr R	*VGCGATSGAMACRTTRCC*		s, 72°C 20 s; 40cycles	

References

1. Fierer N., Jackson J. A., Vilgalys R., Jackson R. B. Assessment of soil microbial community structure by use of taxon-specific quantitative PCR assays. *Appl Environ Microb*. **71**, 4117-4120 (2005).
2. Mori K., Kim H., Kakegawa T., Hanada S. A novel lineage of sulfate-reducing microorganisms: Thermodesulfobiaceae fam. nov., Thermodesulfobium narugense, gen. nov., sp. nov., a new thermophilic isolate from a hot spring. *Extremophiles*. **7**, 283-290 (2003).
3. Chen X. P., Zhu Y. G., Xia Y., Shen J. P., He J. Z. Ammonia‐oxidizing archaea: important players in paddy rhizosphere soil? *Environ Microbiol*. **10**, 1978-1987 (2008).
4. Pester M. *et al.* amoA‐based consensus phylogeny of ammonia‐oxidizing archaea and deep sequencing of amoA genes from soils of four different geographic regions. *Environ Microbiol*. **14**, 525-539 (2012).
5. Poly F., Wertz S., Brothier E., Degrange V. First exploration of Nitrobacter diversity in soils by a PCR cloning-sequencing approach targeting functional gene nxrA. *Fems Microbiol Lett*. **63**, 132-140 (2008).
6. Bru D., Sarr A., Philippot L. Relative Abundances of Proteobacterial Membrane-Bound and Periplasmic Nitrate Reductases in Selected Environments. *Appl Environ Microb*. **73**, 5971-5974 (2007).
7. López-Gutiérrez J. C. *et al.* Quantification of a novel group of nitrate-reducing bacteria in the environment by real-time PCR. *J Microbiol Meth*. **57**, 399-407 (2004).
8. Yan T. F. *et al.* Molecular diversity and characterization of nitrite reductase gene fragments (nirK and nirS) from nitrate- and uranium-contaminated groundwater. *Environ Microbiol*. **5**, 13-24 (2003).
9. Throbäck I. N., Enwall K., Jarvis Å., Hallin S. Reassessing PCR primers targeting nirS, nirK and nosZ genes for community surveys of denitrifying bacteria with DGGE. *Fems Microbiol Lett*. **49**, 401-417 (2004).
10. Braker G., Tiedje J. M. Nitric oxide reductase (norB) genes from pure cultures and environmental samples. *Appl. Environ. Microbiol*. **69**, 3476-3483 (2003).
11. Scala D. J., Kerkhof L. J. Nitrous oxide reductase (nosZ) gene-specific PCR primers for detection of denitrifiers and three nosZ genes from marine sediments. *Fems Microbiol Lett*. **162**, 61-68 (1998).
12. Shaffer B., Widmer F., Porteous L., Seidler R. Temporal and spatial distribution of the nifH gene of N2 fixing bacteria in forests and clearcuts in western Oregon. *Microb Ecol*. **39**, 12-21 (2000).
13. Bach H.-J., Hartmann A., Schloter M., Munch J. PCR primers and functional probes for amplification and detection of bacterial genes for extracellular peptidases in single strains and in soil. *J. Microbiol. Meth*. **44**, 173-182 (2001).

Table S4 Nitrogen functional gene groups used in linear regression analysis.

NO.	Variable	Comment
1	AOA	NH_4^+-N oxidation
2	AOB	NH_4^+-N oxidation
3	nxrA	NO_2^--N oxidation
4	narG	NO_3^--N reduction
5	napA	NO_3^--N reduction
6	nirK	NO_2^--N reduction
7	nirS	NO_2^--N reduction
8	norB	NO reduction
9	nosZ	N_2O reduction
10	apr	mineralization of organic nitrogen
11	nifH	nitrogen fixation
12	(AOB+AOA)	NH_4^+-N oxidation
13	(napA+narG)	NO_3^--N reduction
14	(nirK+nirS)	NO_2^--N reduction
15	AOA/Archaeal16S rRNA	NH_4^+-N oxidation
16	AOA/Bacterial16S rRNA	NH_4^+-N oxidation
17	AOB/Archaeal16S rRNA	NH_4^+-N oxidation
18	AOB/Bacterial16S rRNA	NH_4^+-N oxidation
19	nxrA/Archaeal16S rRNA	NO_2^--N consumption
20	nxrA/Bacterial16S rRNA	NO_2^--N consumption
21	narG/Bacterial16S rRNA	NO_3^--N reduction
22	napA/Bacterial16S rRNA	NO_3^--N reduction
23	nirK/Bacterial16S rRNA	NO_2^--N reduction
24	nirS/Bacterial16S rRNA	NO_2^--N reduction
25	norB/Bacterial16S rRNA	NO reduction
26	nosZ/Bacterial16S rRNA	N_2O reduction
27	nifH/Bacterial16S rRNA	nitrogen fixation
28	apr/Bacterial16S rRNA	mineralization of organic nitrogen
29	(AOB+AOA)/bacterial	NH_4^+-N oxidation
30	(napA+narG)/Bacterial	NO_3^--N reduction
31	(nirK+nirS)/Bacterial	NO_2^--N reduction
32	(AOB+AOA+nxrA)/bacterial	The relative abundance of nitrifying bacteria
33	(narG+napA+nirS+nirK+norB+nosZ)/Bacterial	The relative abundance of denitrifying bacteria
34	AOA/AOB	NH_4^+-N oxidation
35	AOA/nxrA	NO_2^--N accumulation
36	AOB/AOA	NH_4^+-N oxidation
37	AOB/nxrA	NO_2^--N accumulation
38	nxrA/narG	NO_2^--N consumption
39	nxrA/napA	NO_3^--N accumulation
40	nxrA/nirS	NO_2^--N consumption
41	nxrA/nirK	NO_2^--N consumption
42	nxrA/AOA	NO_2^--N consumption

NO.	Variable	Comment
43	nxrA/AOB	NO_2^--N consumption
44	nxrA/(nirK+nirS)	NO_2^--N consumption
45	napA/narG	NO_3^--N reduction
46	napA/nirS	NO_2^--N accumulation
47	napA/nirK	NO_2^--N accumulation
48	nirK/(napA+narG)	NO_2^--N consumption
49	nirS/(napA+narG)	NO_2^--N consumption
50	napA/(nirS+nirK)	NO_2^--N accumulation
51	narG/(nirS+nirK)	NO_2^--N accumulation
52	(narG+napA)/(nirS+nirK)	NO_2^--N accumulation
53	(nirS+nirK)/norB	NO accumulation
54	(nirS+nirK)/nosZ	NO and N_2O accumulation
55	(nirS+nirK)/(narG+napA)	NO_2^--N consumption
56	narG/(narG+napA+nirS+nirK+norB+nnosZ)	Relative share of NO_3^--N reduction in denitrification process
57	napA/(narG+napA+nirS+nirK+norB+nnosZ)	Relative share of NO_3^--N reduction in denitrification process
58	nirS/(narG+napA+nirS+nirK+norB+nnosZ)	Relative share of NO_2^--N reduction in denitrification process
59	nirK/(narG+napA+nirS+nirK+norB+nnosZ)	Relative share of NO_2^--N reduction in denitrification process
60	norB/(narG+napA+nirS+nirK+norB+nnosZ)	Relative share of NO reduction in denitrification process
61	nosZ/(narG+napA+nirS+nirK+norB+nnosZ)	Relative share of N_2O reduction in denitrification process
62	(narG+napA)/(narG+napA+nirS+nirK+norB+nnosZ)	Relative share of NO_3^--N reduction in denitrification process
63	(nirS+nirK)/(narG+napA+nirS+nirK+nosZ)	Relative share of NO_2^--N reduction in denitrification process

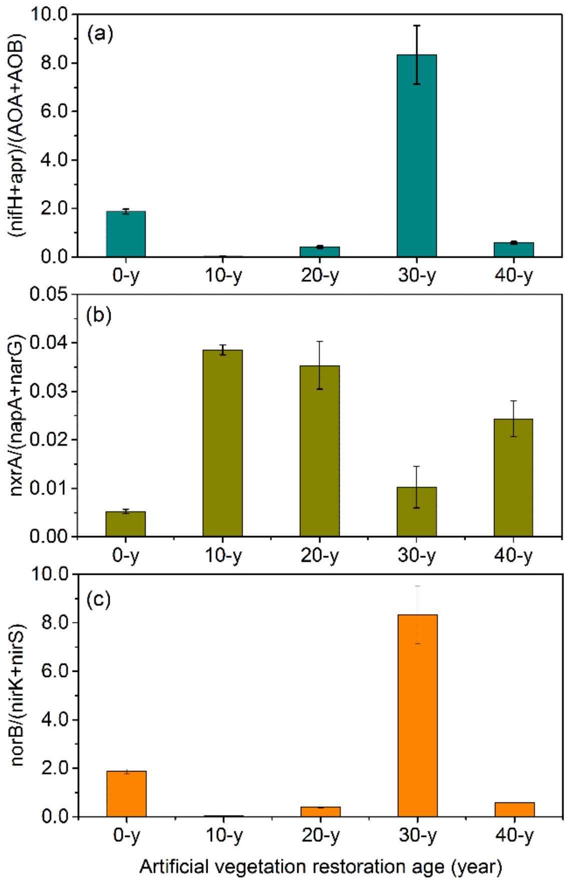

Fig. S1 Effects of the artificial vegetation restoration on gene ratios. (a): (*nifH* + *apr*)/(AOA + AOB), defined as: NH_4^+-N accumulation; (b): *nxrA*/(*napA* + *narG*), defined as: NO_3^--N accumulation; (c): *norB*/(*nirK* + *nirS*), defined as: NO emission; and (d): *norB*/*nosZ*, defined as: N_2O emission. AOA: AOA-*amoA*; AOB: AOB-*amoA*. Data are mean ± standard error (n =3).
